# Rapid and Accurate Differentiation of *Mycobacteroides abscessus* Complex Species by Liquid Chromatography-Ultra-High-Resolution Orbitrap™ Mass Spectrometry

**DOI:** 10.3389/fcimb.2022.809348

**Published:** 2022-03-09

**Authors:** Amol O. Bajaj, E. Susan Slechta, Adam P. Barker

**Affiliations:** ^1^ ARUP Institute for Clinical and Experimental Pathology, Salt Lake City, UT, United States; ^2^ Department of Pathology, University of Utah Health Sciences Center, Salt Lake City, UT, United States

**Keywords:** LC-MS, species differentiation, mycobacteriology, *Mycobacteroides abscessus* (*Mabs*), clinical diagnostics

## Abstract

In this study, a Liquid Chromatography-Mass Spectrometry (LC-MS) method for the identification of clinically relevant *Mycobacteroides abscessus* (*Mabs*) complex organisms is tested using a set of microbial Type strains. This methodology is based on profiling proteins derived from *Mycobacteroides abscessus* complex isolates. These protein profiles are then used as markers of species differentiation. To test the resolving power, speed, and accuracy of this assay four ATCC type strains and 32 recent clinical isolates of closely related *Mabs* species collected at ARUP laboratories (10 clinical isolate strains of *M. abscessus* subsp. *abscessus*, 10 *M. abscessus* subsp. *massiliense*, 2 *M. abscessus* subsp. *bolletii* and 10 *M. chelonae*) were subjected to this approach. Using multiple deconvolution algorithms, we identified hundreds of individual proteins, with subpopulations of these used as species-specific markers. This assay identified 150, 130, 140 and 110 proteoforms with isocratic elution and 230, 180, 200 and 190 proteoforms with gradient elution for *M. abscessus* (ATCC 19977), *M. massiliense* (DSM 45103), *M. bolletii* (DSM 45149) and *M. chelonae* (ATCC 35752) respectively. Taxonomic species were identified correctly down to the species level with 100% accuracy. The ability to differentiate *Mycobacteroides abscessus complex* at sub-species level can in-turn be helpful for patient management. Data analysis showed ~7-17 proteoforms potentially able to differentiate between subspecies. Here, we present a proof-of-principle study employing a rapid mass spectrometry-based method to identify the clinically most common species within the *Mabs* species complex.

## Introduction

Rapidly growing mycobacteria (RGM) are present everywhere in the environment and cause number of infections in humans such as catheter infection, skin and soft tissue infection, pulmonary infection, disseminated infection, etc. ("Diagnosis and treatment of disease caused by nontuberculous mycobacteria. This official statement of the American Thoracic Society was approved by the Board of Directors, March 1997. Medical Section of the American Lung Association" 1997; Daley and Griffith, 2002; Griffith et al., 2007; Colombo and Olivier, 2008). *Mycobacteroides abscessus*, nontuberculous mycobacterium (NTM), has emerged as an important respiratory pathogen in patients with cystic fibrosis (Olivier et al., 2003; Radhakrishnan et al., 2009; Esther et al., 2010; Bryant et al., 2013; O'Sullivan and Sassetti, 2013). *M. abscessus* complex (*Mabs* complex) comprises several closely related species: *M. abscessus*, *M. massiliense*, *M. bolletii, M. chelonae, M. franklinii, M. imunogenum, M. salmoniphilum and M. saopaulense* responsible for human and zoonotic infections (Adékambi et al., 2004; Adékambi et al., 2006; Bryant et al., 2013).

*M. abscessus* has a functional inducible erythromycin ribosome methyltransferase (*erm*) erm(41) gene which causes resistance to macrolides such as clarithromycin, limiting the treatment options of any infection caused by this bacterium (Nash et al., 2009). *M. bolletti* is an uncommon pathogen and is also shown to be highly resistant to clarithromycin (Kim et al., 2008; Adékambi and Drancourt, 2009; Koh et al., 2009).In contrast, *M. massiliense* is susceptible to clarithromycin as it has a non-functional *erm*(41) gene (Kim et al., 2010). Treatment by marcolides is highly effective for *M massiliense* infections. (Koh et al., 2011). *M. chelonae* causes infections of human skin and soft tissues and does not contain the *erm*(41) gene and is therefore susceptible to macrolide treatment. Considering the variations in resistance to the antibiotic therapy and response of a patient to the treatment, it is clinically important to discriminate these closely related species, which will helpful for patient management (Koh et al., 2011; Nakanaga et al., 2011; Harada et al., 2012; Kothavade et al., 2013).

Though various molecular identification methods are available today, *Mabs* complex isolates cannot be distinguished by single gene sequencing like *rpo*B or *hsp65* to differentiate them at the species level (Macheras et al., 2009). Currently, discrimination of these isolated at the species level relies on sequencing multiple genes such as *erm*(41), 23S rRNA, *rpoB*, *hsp65*, *gnd*, *glpK*, *secA* and *sodA* (Sassetti et al., 2003; Adékambi and Drancourt, 2004; Nash et al., 2009; Kim et al., 2010; Koh et al., 2011). However, this process is quite lengthy which delays the results and is too expensive and complex to be performed in most clinical microbiology laboratories.

Identification of micro-organisms using MALDI-TOF mass spectrometry (MS) has revolutionized the practice of clinical microbiology by decreasing the cost and turnaround time of identification, at the same time increasing test accuracy. However, intrinsic limitations in the analytic resolving power of MALDI-TOF often limit its ability to discriminate closely related organisms (Pignone et al., 2006; Lotz et al., 2010; El Khéchine et al., 2011; Saleeb et al., 2011; Mather et al., 2014). An important example is the failure to differentiate certain species and subspecies of mycobacterium (e.g., *Mabs* complex, *Mtb* complex) which is critical for patient’s management (Griffith et al., 2015). While MALDI-TOF MS can differentiate *M. abscessus* from *M. chelonae*, it cannot separate *M. bollettii* and more importantly *M. massilense* from *M abscessus*. Moreover, the gold standard for AFB identification (16s RNA sequencing) also fails to differentiate these organisms. Also, we have previously demonstrated the use of ultra-high-resolution mass spectrometry technique for identification of closely related mycobacterium tuberculosis complex species but no other report yet outlines this technique for Mabs complex identification (Bajaj et al., 2021). In order to achieve a rapid and highly specific test for the identification of mycobacteria to the species level a high-resolution MS approach was developed. In this study the microbial extracts were separated using micro (nano) flow liquid chromatography and then a high-resolution orbitrap mass spectrometer was used to analyze the separated proteins. The workflow consisted of cell sonication, solid-phase extraction (SPE) purification, mass spectrometry, and computational algorithms to achieve the differentiation of these organisms with high identification accuracy. The aim of the present study was to provide a proof-of-principle experiment employing orbitrap liquid chromatography mass spectrometry (LC-MS) for differentiation of Mabs complex species. This is the first study in which differentiation of *Mabs* complex at a species level is achieved. By employing this mass spectrometry driven approach there is a potential to discriminate all types of closely related *Mabs* pathogens by this method.

## Materials and Methods

### Selection of Strains for Identification

Mycobacterium strains belonging to the species M. abscessus, M. massiliense, *M. bolletti* and *M. chelonae* were selected as representatives for the Mabs complex. Though M. franklinii, M. imunogenum, M. salmoniphilum and M. saopaulense are also part of the Mabs complex, we excluded them in this study since their frequency as causative agents of zoonotic human infections is rare and their partially unclarified taxonomic status. To anchor our species classification, ATCC and DSMZ Type strain isolates were selected for analysis [*M. abscessus* (ATCC 19977), *M. massiliense* (DSM 45103), *M. bollettii* (DSM 45149) and *M. chelonae* (ATCC 35752)]. In addition, 32 recent clinical isolates obtained by ARUP laboratories (10 clinical isolate strains of *M. abscessus*, 10 *M. massiliense*, 2 *M. bolletii* and 10 *M. chelonae*) were analyzed to assess downstream assay robustness. All clinical isolates were identified by quantitative (real-time) PCR and melt curve analysis with a multiplex probe set **(** Pounder et al., 2010 **)** prior LC-MS data acquisition. All strains of recent clinical origin were collected throughout the year 2019 at ARUP laboratories (Salt Lake City, UT), according to protocols approved by the University of Utah Institutional Review Board (IRB) with no additional metadata being available (double blinded clinical identifiers).

### Chemicals and Reagents

The Optima LC-MS grade Water (H2O), Optima LC-MS grade acetonitrile (ACN), Optima LC-MS grade formic acid (FA) were purchased from Fisher Scientific (Fair Lawn, NJ). 70% Ethanol was purchased from in-house reagent laboratory and 7H11 agar plates from Hardy Diagnostics (Santa Maria, CA).

### Sample Preparation

#### DNA Extraction, PCR and Sequencing

Genomic DNA was extracted using in-house developed Chemagic MSM 1 protocol for nucleic acid extraction and its concentration was measured using Qubit dsDNA BR Assay Kit according to the manufacturer’s protocol. Chromosomal DNA was then fragmented, purified, and then subjected to PCR. Double-sided size selection was performed and then the library was amplified and purified. After quality determination the library was sequenced using Iontorrent S5 (Thermo Scientific). Identification was performed by blasting initial sequences to nucleotide, then reference database of Type strains that covered the area of gene that was amplified was constructed and then clinical sequences were compared to the references *via* blast. Identifications were made based on consensus answers.

#### Preparation of Whole Cell Extracts (WCE) for *M. abscessus* (Mabs) Complex Species

The ATCC/DSMZ type strains and clinical isolates of *M. abs* complex were cultured on 7H11 agar plates for 4-5 days in a CO_2_ incubator maintained at 37°C. Approximately 1 µL loopful of colonies was transferred into sonication vials (Thermo Fisher Scientific, proprietary). The cells were then dispensed in a pre-incubation solution containing alcohol (Thermo Fisher Scientific, proprietary) at room temperature (RT, 20-25°C). After a short centrifugation step (12,000 x g for 2 minutes at RT) the supernatant was discarded and to the pellet 100 µL of incubation solution containing formic acid and acetonitrile (Thermo Fisher Scientific, proprietary) was added. The cell lysate was incubated for 20 minutes (vortexed once at 10 minutes for 2 seconds). Sonication was then performed for one minute at 50% amplitude, then 100 µL dilution buffer containing acetonitrile (Thermo Fisher Scientific, proprietary) was added to the lysed cells and centrifuged at 12,000 × g at RT for 5 minutes. The supernatant was collected in low protein binding Eppendorf (LBE) tubes and can be stored at -80°C until its LC-MS analysis is performed.

#### SPE Method

The WCE if stored at -80°C was thawed to RT and then diluted with SPE buffer-1 (Thermo Fisher Scientific, proprietary) before SPE cleanup. Conditioning and equilibration of the Reverse Phase monolith (RP4H) SPE tips (Thermo Fisher Scientific, proprietary) was performed by placing them in a 96 well-plate with 50 µL SPE buffer-1 and SPE buffer-2 (Thermo Fisher Scientific, proprietary), respectively. The plates were then centrifuged at 2000 × g for 2 minutes at RT. Diluted WCE (50 µL) was loaded into the SPE tips and centrifuged. Washing of the tips was carried out with 50 µL of proprietary SPE buffer-3 (Thermo Fisher Scientific, proprietary). The tips were then placed in liquid chromatography (modified Easy-1000, Thermo Fisher Scientific) autosampler plate for further online elution and mass spectrometric (MS) analysis. For negative control samples tips designated as blanks were prepared in a similar way as described above, where instead of cell lysate water was added to the SPE tips.

#### Analysis Using LC-MS

**Figure 1:**
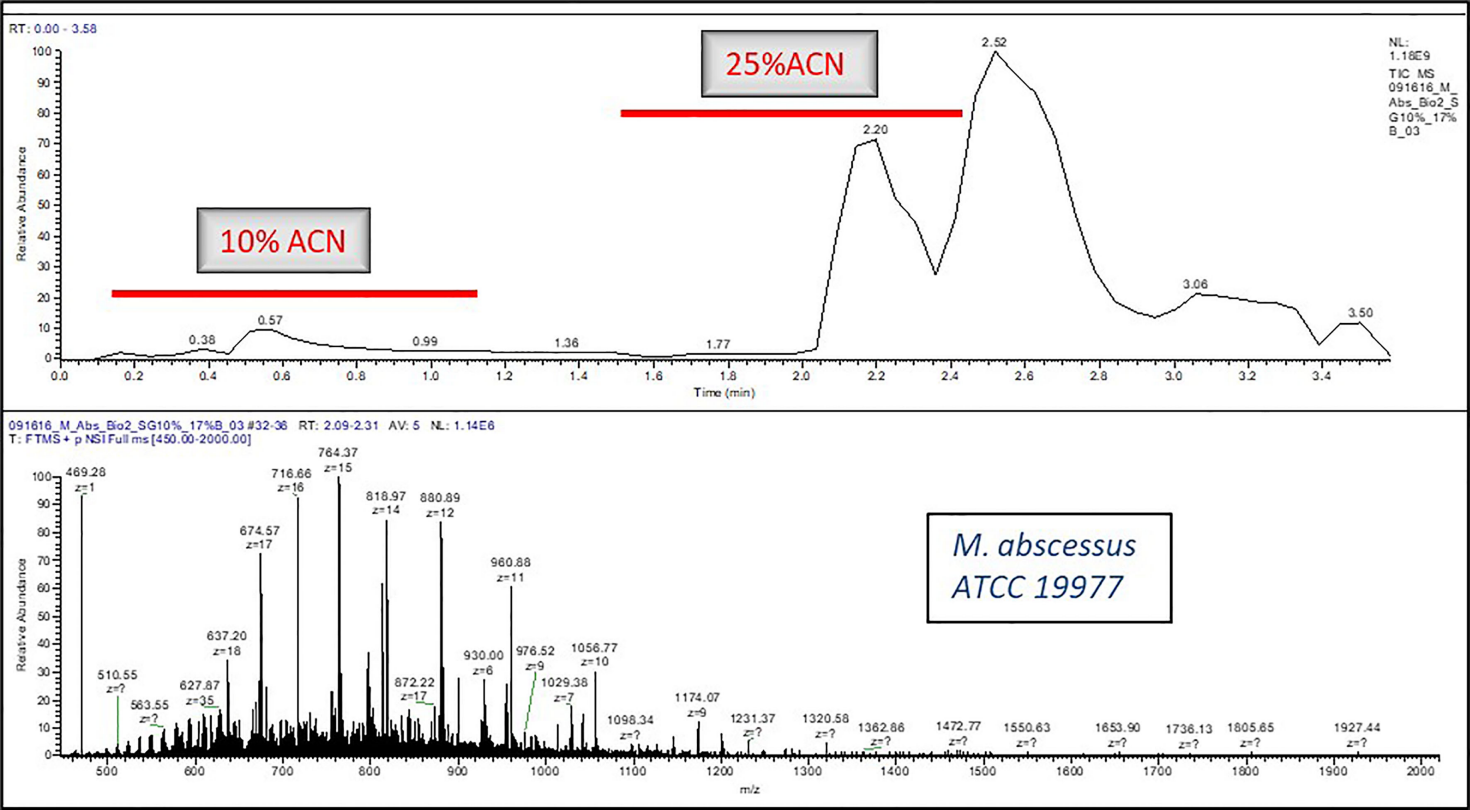
A typical LC-MS chromatogram of Isocratic Elution for e.g., *Mycobacteroides abscessus ATCC19977*.

**Figure 2:**
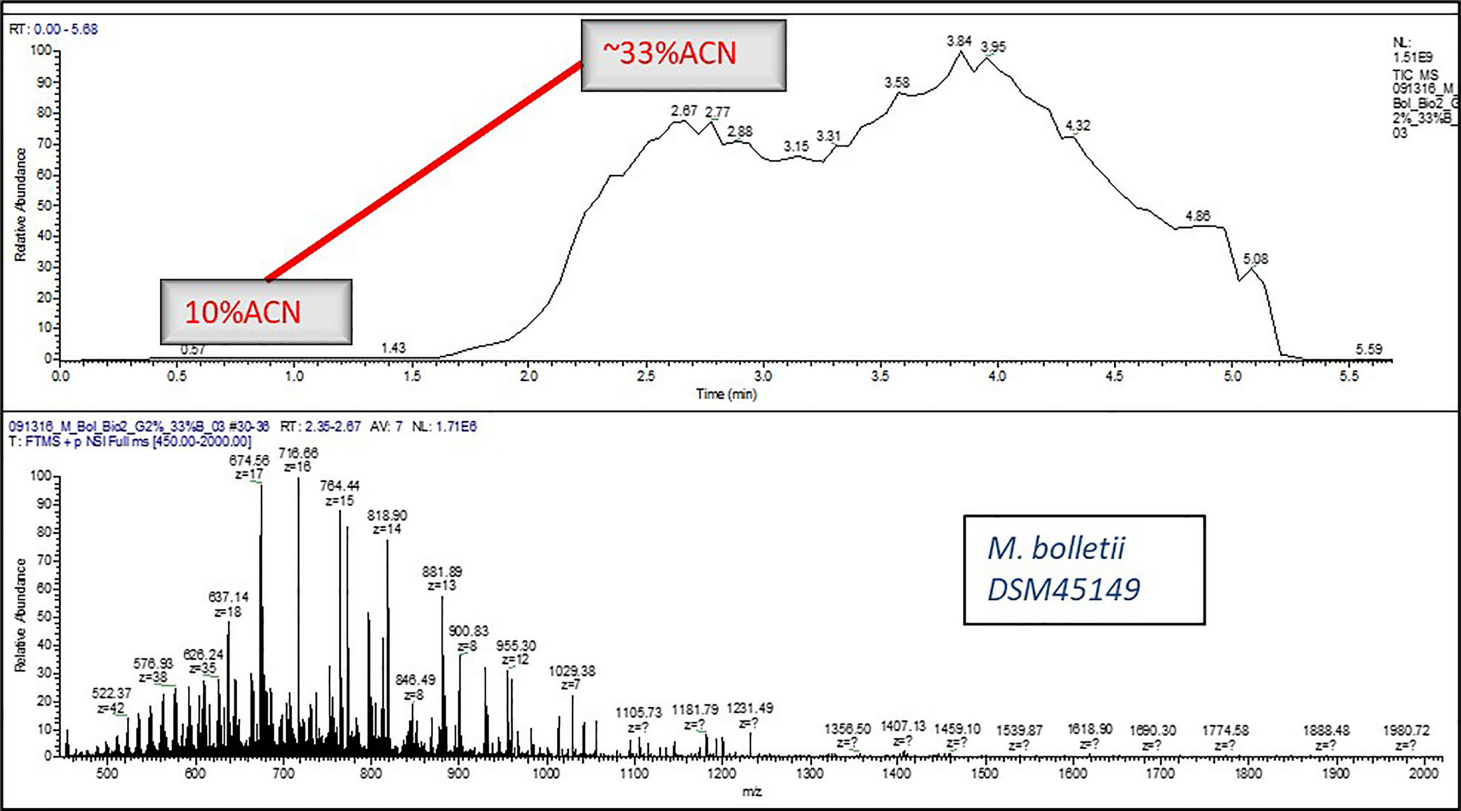
A typical LC-MS chromatogram of Gradient Elution for e.g., *Mycobacteroides bolletii DSM45149*.

A micro flow LC-MS system was used to perform all the experiments. A Q Exactive HF Orbitrap mass spectrometer (Thermo Fisher Scientific, San Jose, CA) was used as a detector which was hooked with the liquid chromatography (LC) system. The 0.2% FA, 10% ACN in H2O was the mobile phase A composition and the 0.2% FA in ACN was the composition for mobile phase B. Elution was carried out as per the following scripts: 1. **Isocratic Elution:** After flow stabilization at 4 µL/min for 2%B, 2%B was run for approximately 2 min, eventually stepping-up the gradient to 17%B for 2 more minutes (**Figure 1**), 2. **Gradient Elution:** After flow stabilization at 4 µL/min for 2%B, percent of B was increased to 33% B in 5 min (**Figure 2**).

Xcalibur software version 3.0 (Thermo Fisher Scientific) controlled the LC-MS. The samples were ionized in the positive ionization mode (+ESI) within the MS. The general mass spectrometric conditions were: spray voltage - 2.0k V, capillary temperature - 325°C, and S-lens RF level – 65 maximum injection time - 200 millisecond and number of microscans was set to 40. 3e^6^ was used as the ACG target, and the MS resolution was set to 120,000. A 0.2 of trapping gas pressure was used with the MS set to intact protein mode and C-trap charge detection was set OFF. The mass range from 450 to 2000 *m*/*z* for full scan mode was used for analysis of proteins.

#### Data Analysis

**Figure 3:**
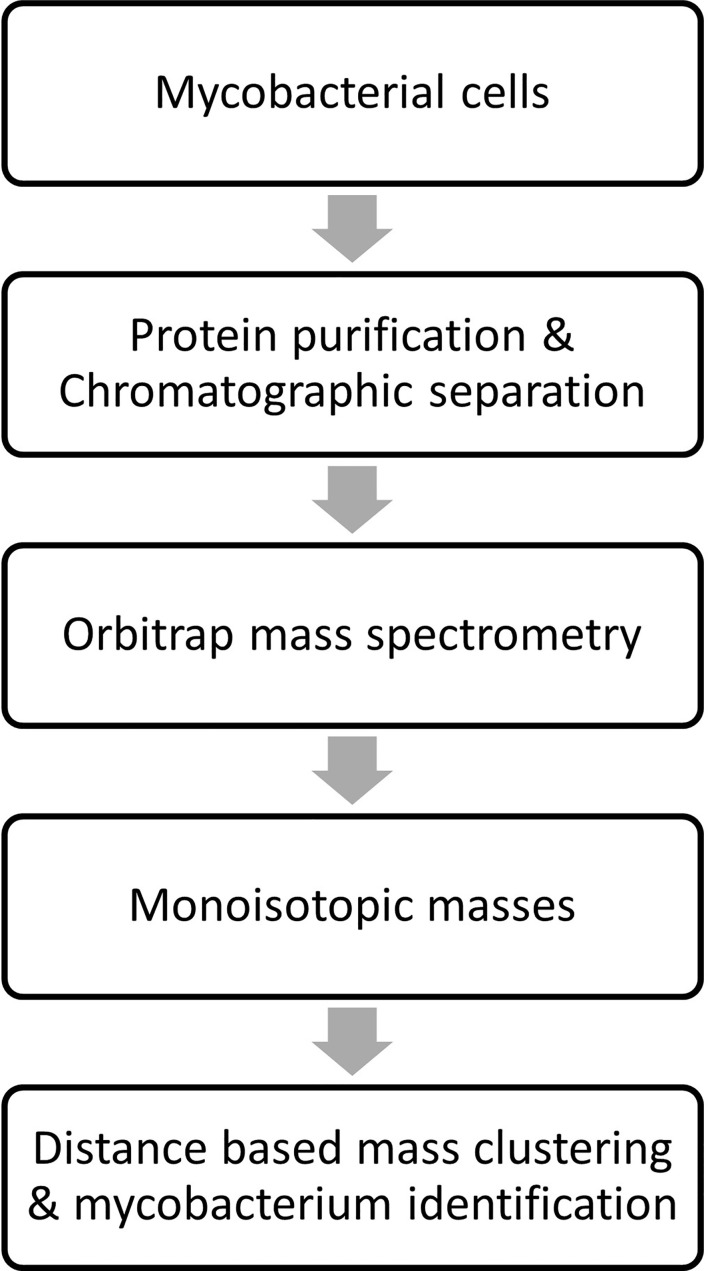
Overview of sample processing, data acquisition and analysis steps employed in stepwise analysis of LC-MS Orbitrap data.

The monoisotopic masses (i.e., list of proteoforms) were produced from the raw data by deconvolution using proprietary algorithm. The in-house algorithms were used for building the database and the generated data from the clinical isolates was then processed against it for identification of mycobacterium species. An overview of sample processing, data acquisition and analysis steps employed in stepwise analysis of LC-MS orbitrap data is shown in **Figure 3**. Two technical replicates were run for each strain to construct the database and subsequently identify the species. Each ATCC and DSMZ strain in the analysis was also analyzed in six replicates (two technical replicates from three biological replicates) for the assessment of technical reproducibility of the sample extraction and analysis. The mass spectra generated were processed by Thermo Fisher proprietary software to deconvolute raw spectra into monoisotopic protein masses between 5 and 40 kDa. A list of consensus markers was aligned and constructed from these monoisotopic masses. Top 200 consensus markers were selected using one-way ANOVA that had the most predictive value for the differentiation of the species. The Thermo Fisher Scientific proprietary distance-based clustering algorithm was used for predicting the species. The robustness of species prediction was reproduced by leave-one-out iterations where each sample was tested against the constructed database without data for the same strain (training and test dataset). The data will be made available on reasonable request.

## Results

### MLST

Sequence analysis of the *rpoB* and *hsp65* genes of the 33 clinical isolates of *M. abscessus* complex led to identification of 14 *M. abscessus* subsp. *abscessus* isolates, 10 *M. abscessus* subsp. *massiliense* isolates, 2 *M. abscessus* subsp. *bolletii* isolates and 6 were multiple species slash calls and 1 failed sequencing.

### Inactivation

All tested formic acid and acetonitrile combination solvents were able to inactivate mycobacteroides at selected solvent incubation time intervals (10 min, 20 min, 30 min and 1 hr) but formic acid and acetonitrile solvent (proprietary ratio) for 20 minutes incubation was chosen as preferred method. An addition of 70% ethanol before this step was tested for inactivation and was included in the sample preparation process. No growth of mycobacteroides was observed on 7H11 agar plates even after 2 weeks of incubation at 37°C confirming inactivation of these rapidly growing mycobacteria.

### SPE Clean-Up and SALC Method Development

WCE was prepared by lysing a few colonies of the microbe harvested using specialized loop (Thermo Fisher Scientific, proprietary) from 7H11 agar plates (Hardy Diagnostics) in the Thermo Fisher proprietary buffer using sonication (Thermo Fisher Scientific, proprietary). When measured using Qubit Protein Assay (Thermo Fisher Scientific), protein concentration of the WCE was found to be 0.2-0.3 µg/µl. Different SPE materials (data not shown) like C4 (Waters Corp.), POROS R2 (Glygen Corp.) and RP4H (Thermo Fisher Scientific) were tested for the WCE clean-up but comparatively RP4H was found to be effective for our purpose.

Different isocratic flow (20%, 25%, 30%, 40%, 50% and 60% ACN- data not shown) for different acquisition time (1 min, 2 min and 4 min) and gradient flow (5min and 8 min) was tested but 25% ACN stepwise elution for 3 min and gradient elution for 5 min was found to work best for yielding sufficient proteoforms to be considered for the data analysis. In addition, the wash step after loading sample on SPE was tested for 4% ACN, 10% ACN (data not shown) and 0.2% FA in water wash. The 0.2% FA in water wash was more effective than the other two solvents considered for washing SPE.

### Data Analysis

Approximately 100-150 proteoforms were identified using isocratic elution; the number of identified proteoforms increased ~1.5-2 fold with a gradient elution.

### Reasoning and Verification of LC-MS Proteome Motif

**Figure 4:**
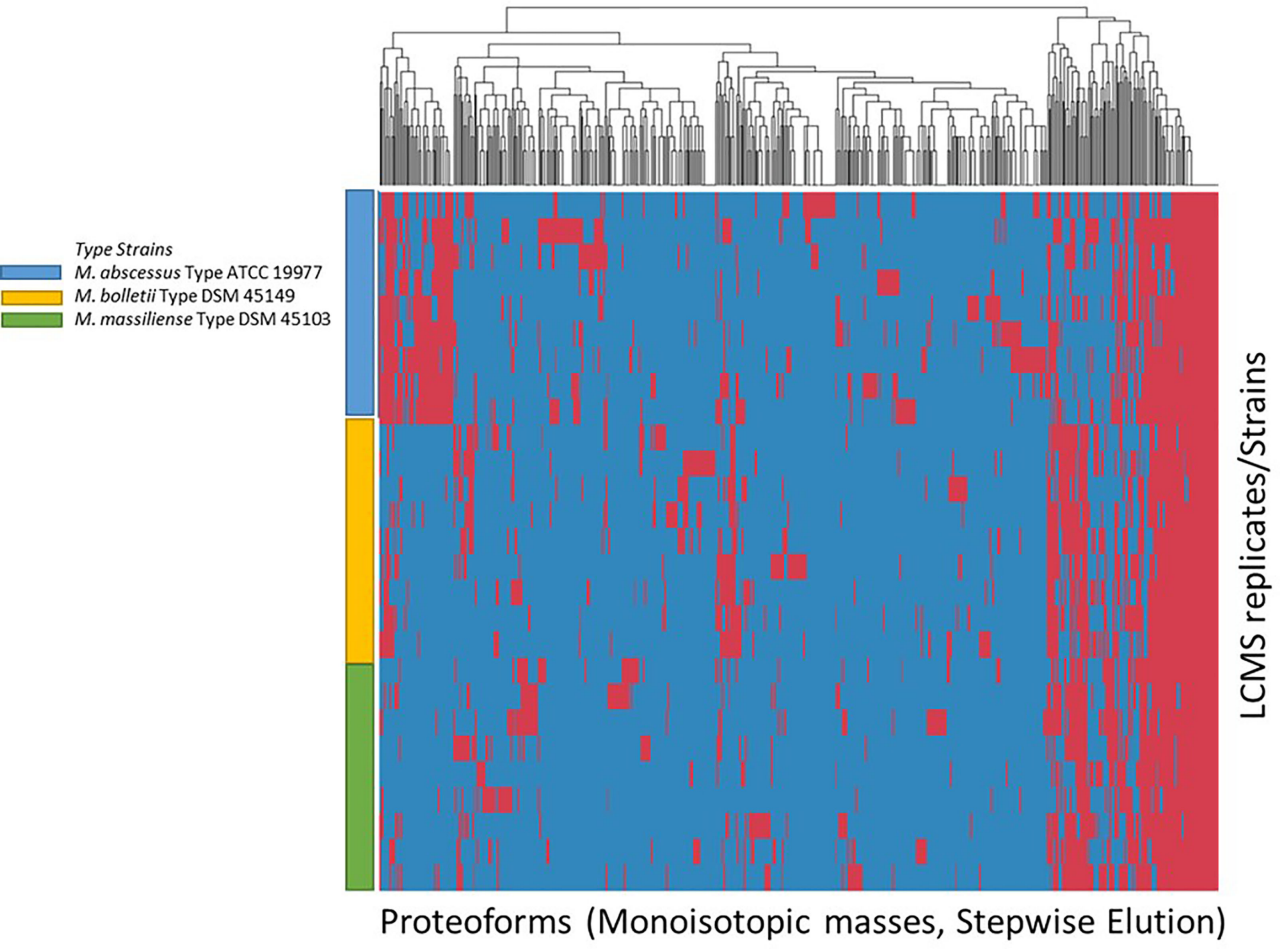
Clustering of Proteoforms visualized as heat map for *M. abscessus*, *M. massiliense* and *M. bolletii* using stepwise elution with top 500 proteoforms plotted.

**Figure 5:**
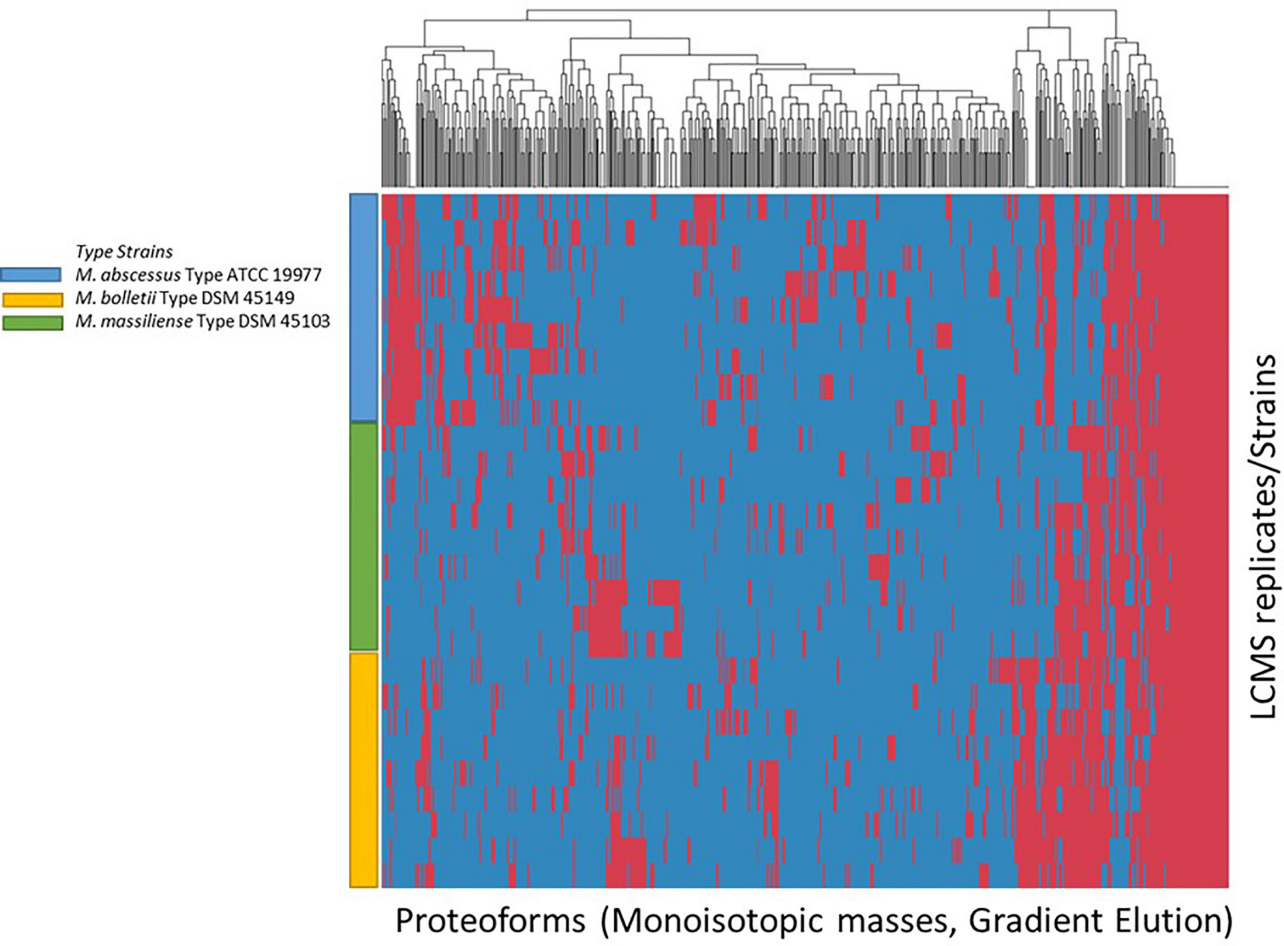
Clustering of Proteoforms visualized as heat map for *M. abscessus*, *M. massiliense* and *M. bolletii* using gradient elution with top 500 proteoforms plotted.

The list of consensus markers was constructed by aligning the masses of the deconvoluted proteoforms from each species to determine if different *Mabs* complex species show distinctive MS spectrum profiles. The deconvoluted spectrum profiles that generated the proteoforms was used to determine common and uncommon proteoforms (**Figures 4**, **5**). The masses present in most replicates of a given species and primarily absent from replicates of other species were presented to be unique. To verify whether different *Mabs* complex strains show similar mass spectral profiles, we analyzed 32 clinical isolates of *Mabs* complex with the outlined LC-MS analysis workflow (**Figure 3**). The mass spectrum profiles obtained from the ATCC and DSMZ type strains were consistent with all the clinical isolates within a species, and they displayed a similar pattern (data not shown), which comprises common proteoforms ranging from 5 to 40 kDa. The similar elution profiles and identified proteins from the strains shows that there are consistent species-specific proteome patterns.

### Differentiation of Mabs Complex Species

**Figure 6:**
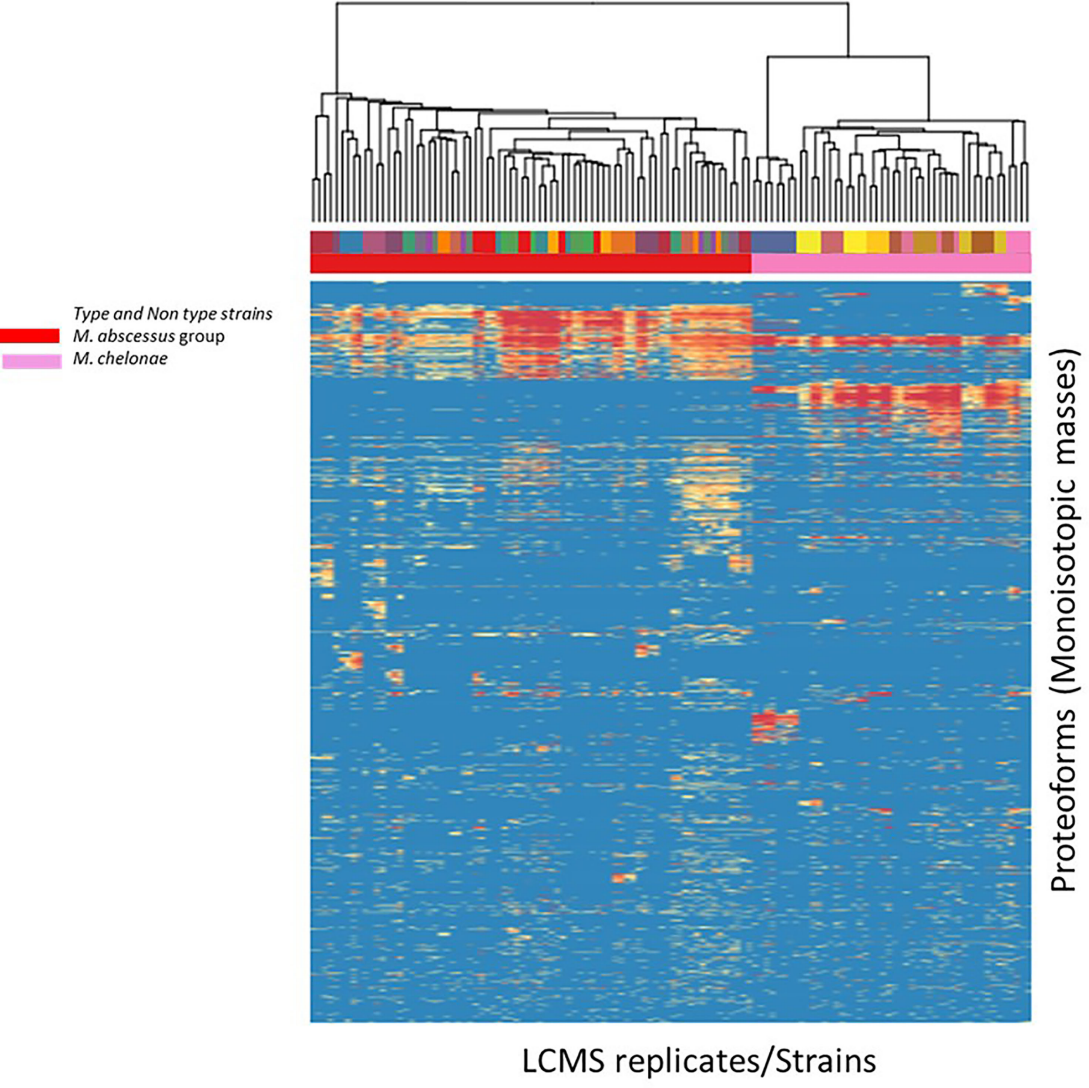
Heat map generated for *M. abscessus* and *M. chelonae* analyzed indicating top-200 discriminatory proteoforms. Horizontal bar on top, indicates the color assignment for the type and non-type strains used in the study, horizontal bar below indicates species assignment alone for visual clarity (*M. abscessus group* (red) and *M. chelonae* (pink)). Mass spectrometry data is here represented as a heatmap, where rows represent individual monoisotopic masses (deconvoluted protein masses) and columns individual strains (technical LC-MS replicates for each strain were merged to a single leaf in the horizontal dendrogram) of the study cohort. Data has been converted to a binary state, where 1 (red) encodes presence of a protein mass in each strain (species) and 0 (blue) encodes absence of it. The representation indicates proteoform overlap and non-overlap between strains among all species due to their closeness at species level. Unique proteoforms detected among individual species specifically enable the classification down to species level (Specific number of exclusive red blocks for a given taxon). Red block of proteoforms is shared between the species of interest and is non-species specific (conserved). The dendrogram shows that the *M. abscessus* and *M. chelonae* clinical isolates are split in two separate branches, allowing discrimination of these fundamentally different strains.

We evaluated the ability of the MS spectrum profile and the common proteoforms obtained from different species within *Mabs* complex could aid in differentiating the species within the complex. Based on the different expression of unique masses in the species-specific protein profiles, we developed species-specific protein databases capable of rapidly differentiating *Mabs* complex at species level accurately. As represented in **Figures 4** and **5**, *M. abscessus* and *M. massilliense* are differentiated based on unique protein profiles. The type-strain of *M. bolletti* displays enough unique proteoforms to cluster separately based on our clustering algorithm. We were also able to differentiate *M. abscessus* group from *M. chelonae* as show in **Figure 6**. Based on the different expression of species-specific protein profiles, we were capable of rapidly differentiate the *Mycobacterium abscessus* complex to the species level. Furthermore, differentiating proteoforms may serve as potential biomarkers in identification of these organisms in patient samples in additional confirmatory studies using clinical specimens. Thus, LC-MS technology could be developed to become a first-line platform in the rapid and accurate identification of *Mabs* complex species in routine clinical microbiological laboratories.

### Reproducibility

Around 3 biological replicate and a total of 9 technical MS runs of each species of the complex was carried-out and the proteoforms obtained from each run when compared to each other were consistent between replicates.

## Discussion

This is the first study to our knowledge which is focused on species level classification of *M. abscessus, M. massiliense, M. bolletii and M. chelonae*, using a high-resolution mass spectrometry. The important finding from our study is discriminating species with close to 100% accuracy quickly of time using proteoforms from each species and with both isocratic as well as gradient elution methods. MALDI-TOF MS methods have been applied to fungal, bacterial and mycobacterial identification and shown to be a reliable technique in routine laboratory. Use of MALDI-TOF MS for discrimination of mycobacteria is relatively rapid and accurate, however, this technique cannot differentiate all *Mabs* complex species. In this study, we demonstrated that ESI-Orbitrap LC-MS could be used in the rapid and accurate identification of *Mabs* complex species. Ionizing analytes to produce multiple charge states gives a distinct advantage to using ESI, bringing the mass to charge ratio of larger proteins into the window of the mass spectrometer range. The increased discriminatory power for speciation between highly similar species was achieved using the higher mass proteins which leads to the improved success of this approach. A top-down proteomics workflow can help to study protein identification and further characterization which was not performed in this study. *M. abscessus* species is an important pathogen worldwide, algorithm robustness and interlaboratory reproducibility are essential when identifying these organisms in a clinical setting. All the clinical isolates were from USA, which might introduce bias. The algorithm therefore would benefit from testing in a larger, worldwide collection of isolates to check biogeographic MS profiles for a given species which might produce similar results.

In this study, we show potential for using LC-MS and machine learning for species level differentiation of *Mabs* complex organisms with the high success of classifying the species. The large number of spectra generated from these strains are used to produce classification models. The goal was that the species under evaluation can be classified accurately using a common signature among spectra for each of the identified taxa. The mass spectra that generated proteoforms in the approximate mass range of 5 – 40 kDa was used by the algorithm. The differentiation of clinically relevant mycobacterial species and clinical isolates at a species level was carried out using the protein profile from the mass range selected above with high accuracy. In general, the molecular taxonomy of mycobacterium is dynamic, and species boundaries are highly arbitrary. In our approach we precisely outline the possibility to identify taxonomic entities (such as specific strains) that correspond to clinical outcome (treatment decision) using the mass spectrometry-based approach.

## Conclusion

The described assay is the first study of the use of LC-ESI-MS to demonstrate the accurate identification of *M. abscessus* complex isolates to the species level because its analytical performance level is higher than that of MALDI-TOF. The assay can rapidly, and accurately distinguish closely related *M. abscessus* complex organisms to a species level identification. The complex species are closely related member of *M. abscessus* complex but diverse pathogenetically and in their available treatment options. To improve diagnosis, treatment and understand epidemiology of the pathogens an effective and accurate way to distinguish them is really required. The proprietary Thermo Scientific algorithms showed a potential to recognize individual strains. Additional research is required for comprehensive investigation of protein profiles and identifying unique protein patterns from these *Mycobacterial species* which will provide us more insight into pathogenicity related to these microbes. Additional work is also required to understand proteomic diversity of atypical *Mabs* isolates, since reliable identification must capture all clinically relevant variants of a given species for accurate microbial diagnostics. Larger sample sizes, evaluation in different growth media and an improved protocol will provide confirmation of this first proof of principle study of discriminating *M. abscessus* complex species.

## Data Availability Statement

The raw data supporting the conclusions of this article will be made available by the authors, without undue reservation.

## Author Contributions

AOB have drafted the work. AOB and APB designed research. AOB performed experimental work, data acquisition, and analysis. AOB, ESS and APB conducted review and editing. APB provided funding acquisition, project administration, and resources. All authors contributed to manuscript revision, read, and approved the submitted version.

## Funding

This study received funding from Thermo Fisher Scientific. The funder was not involved in the study design, collection, analysis, interpretation of data, the writing of this article or the decision to submit it for publication.

## Conflict of Interest

The authors declare that the research was conducted in the absence of any commercial or financial relationships that could be construed as a potential conflict of interest.

## Publisher’s Note

All claims expressed in this article are solely those of the authors and do not necessarily represent those of their affiliated organizations, or those of the publisher, the editors and the reviewers. Any product that may be evaluated in this article, or claim that may be made by its manufacturer, is not guaranteed or endorsed by the publisher.
